# A new genus of Cletopsyllidae (Copepoda, Harpacticoida) from Gulf of Mexico

**DOI:** 10.3897/zookeys.391.6903

**Published:** 2014-03-19

**Authors:** Hyun Woo Bang, Jeffrey G. Baguley, Heejin Moon

**Affiliations:** 1 Department of Biology, University of Nevada Reno, Reno, Nevada 89557, USA

**Keywords:** Harpacticoida, Cletopsyllidae, *Pentacletopsyllus* gen. n., meiofauna, Gulf of Mexico

## Abstract

A new genus and new species of the family Cletopsyllidae Huys & Willems, 1989 from deep-sea sediment in the Gulf of Mexico, are reported and fully described and illustrated. The new genus *Pentacletopsyllus* (type species: *P. montagni* **sp. n.**) can be distinguished from the four known genera of the family by antennule segmentation, length ratio of first and second endopodal segments of P1, and armature pattern on P5 exopod. It also differs from its sister genera by the rostrum being bifid at the tip, third segment of the female antennule having a smooth posterior margin, the baseoendopod of P5 with biarticulate outer setophore bearing basal seta, and female caudal rami without lobate expansion. A revised key to species of the family Cletopsyllidae Huys & Willems, 1989 is provided.

## Introduction

The Gulf of Mexico is a large semi-enclosed oceanic basin surrounded by the North American continent and the island of Cuba. The continental slope of the northern Gulf of Mexico is topographically complex and shows a high species diversity of benthic fauna (Haedrich et al. 2008). Baguley et al. (2006) estimated the harpacticoid species richness of the northern Gulf of Mexico to be approximately 2200 species with a maximum diversity found at mid-slope water depths of 1200–1500 m. Harpacticoid diversity in this area is thought to be maintained by both small scale heterogeneity and large scale food supply mechanisms (Baguley et al. 2006). In total, Baguley et al. (2006) recorded 696 species of benthic harpacticoids, belonging to 175 genera and 22 families.

The family Cletopsyllidae includes only four genera and 11 species, which are rare in marine benthic habitats (Boxshall and Halsey 2004). However, Cletopsyllidae does have a broad global distribution and has been found in the Indian Ocean (*Retrocalcar secundus* (Nicholls, 1945); *Cletopsyllus bacescui* Marcus, 1976; *Bathycletopsyllus hexarthra* Huys & Lee, 1999), the Pacific Ocean (*Retrocalcar sagamiensis* (Itô, 1971); *Isocletopsyllus maximus* Song, Kim & Hwang, 2010), the Atlantic Ocean and the Caribbean Sea (*Cletopsyllus papillifer* Willey, 1935; *Retrocalcar brattstroemi* (Geddes, 1981); *Cletopsyllus rotundifera* Fiers, 1986), and the Mediterranean Sea (*Isocletopsyllus tertius* (Por, 1964); *Isocletopsyllus quartus* (Soyer, 1966); *Isocletopsyllus sardus* Addis, Floris & Carcupino, 2011).

The subfamily Normanellinae was elevated to family level by Huys and Willems (1989), and they established two subfamilies, the Normanellinae and Cletopsyllinae. Subsequently, Huys and Lee (1999) raised the subfamily to the family Cletopsyllidae and it was divided into four genera, the type genus *Cletopsyllus* Wiley, 1935, and three new genera *Bathycletopsyllus*, *Isocletopsyllus* and *Retrocalcar*. At that time, they considered *Pseudocletopsyllus* Vervoort, 1964 to be a *genus inquirendum* in Cletopsyllidae because of the inadequate description of the type species *Pseudocletopsyllus spiniger*, which was very similar to the copepodid V of *Retrocalcar sagamiensis* (Itô, 1971).

During a recent deep-sea benthic survey to assess impacts of the Deepwater Horizon oil spill in the northern Gulf of Mexico, a new genus and species of Cletopsyllidae was collected. Recent investigations have uncovered significant benthic community impacts from the Deepwater Horizon (Montagna et al. 2013), stressing the need to more completely describe and understand the biodiversity and community structure of the region. Here, we describe the new genus and species based on the newly collected specimens and provide an updated key to genera and species of Cletopsyllidae.

## Material and methods

Samples were collected from the northern Gulf of Mexico in May/June 2011 as part of the Deepwater Horizon Natural Resource Damage Assessment (NRDA) follow-up cruise aboard the *R/V Sarah Bordelon*. Sediments were sampled with an Osil multicorer and were fixed with buffered formalin and stained with Rose Bengal. Meiofauna was extracted from sediments by Ludox isopycnic centrifugation (Burgess 2001). Harpacticoids were sorted and enumerated under a Leica S8APO dissecting microscope, and stored in 70% ethanol.

Specimens were dissected in lactic acid and the dissected parts were mounted on slides in lactophenol mounting medium. Preparations were sealed with transparent nail varnish. All drawings have been prepared using a camera lucida on a Leica DM 2500 differential interference contrast microscope. Specimens are deposited at the Smithsonian National Museum of Natural History.

The descriptive terminology is adopted from Huys et al. (1996). Abbreviations used in the text are: A1, antennule; A2, antenna; ae, aesthetasc; exp, exopod; enp, endopod; P1–P6, first to sixth thoracopod; exp (enp)-1 (2, 3)to denote the proximal (middle, distal) segment of a ramus . Scale bars in figures are indicated in μm.

## Results

### Order Harpacticoida Sars, 1903
Family Cletopsyllidae Huys & Willems, 1989

#### Pentacletopsyllus gen. n.

http://zoobank.org/F4DAC7B8-1F8F-4F98-BEE7-8FD00EDDED7D

http://species-id.net/wiki/Pentacletopsyllus

##### Diagnosis.

Cletopsyllidae. Body elongated. Cephalothorax and other somites with numerous spinous processes at margin. Rostrum very prominent, triangular, with bifid tip in its apical portion, with pair of subapical sensilla. Genital and first abdominal somites completely fused forming double-somite. Anal operculum well developed. Caudal rami cylindrical, with 7 setae; setae I-II small, closely set; seta III subapical, setae IV-V bipinnate, seta V longest, seta VI bare, Seta VII tri-articulate at base. Sexual dimorphism in antennule, P2 endopod, P5 and P6.

Antennule 5-segmented in female, 7-segmented and subchirocer in male. Antenna 3-segmented, comprising coxa, allobasis and free endopodal segment; exopod 1-segmented with 2 setae. Mandible with biramous palp; basis with 3 pinnate setae; endopod with 1 lateral and 3 distal setae; exopod 1-segmented with 1 seta. Maxillule with strongly developed praecoxal arthrite, with 2 setae on anterior surface and 9 spines/setae around distal margin; coxal endite with 1 seta and 1 spine; basis with 4 spines/setae; endopod represented by 1 seta; exopod with 2 setae. Maxilla with 3 endites; allobasis drawn out into claw; endopod minute, bearing 3 setae. Maxilliped subchelate, with 3 pinnate setae; basis asetose; endopod drawn out into long, curved, pinnate claw with 1 long bare seta and 1 short accessory seta at base.

P1 basis with outer seta and inner spine; exopod 3-segmented, exp-1 with long outer spine, exp-2 with inner seta, exp-3 with 4 elements; endopod 2-segmented and prehensile, enp-1 as long as enp-2.

P2-P4 with outer spine or seta on basis. Exopods 3-segmented, exp-3 with 3 outer spines; endopod 2-segmented, enp-1 small, enp-2 elongate. Male P2-enp modified; inner apical seta fused to segment forming small apophysis. Armature formula:

**Table:** 

	Exopod	Endopod
P2	0.1.123	1.421
P3	1.1.223	1.321
P4	1.1.223	1.321

P5 of both sexes with separate baseoendopod and exopod. Basal seta on setophore; endopodal lobe triangular with 5 bipinnate setae in female and 3 bipinnate setae in male; exopod elongated with 5 setae in female and 4 setae in male. Male P6 asymmetrical, functional member represented by small plate, opposite member fused to genital somite; each leg with 1 bare seta.

##### Type and only species.

*Pentacletopsyllus montagni* gen. et sp. n.

##### Etymology.

The generic name is derived from the Greek *penta*, meaning five, and refers to the 5-segmented female antennule.

#### Pentacletopsyllus montagni sp. n.

http://zoobank.org/3CD1F4AE-9B8D-422F-A863-4683A3BA1EDC

http://species-id.net/wiki/Pentacletopsyllus_montagni

**Figure 1.**
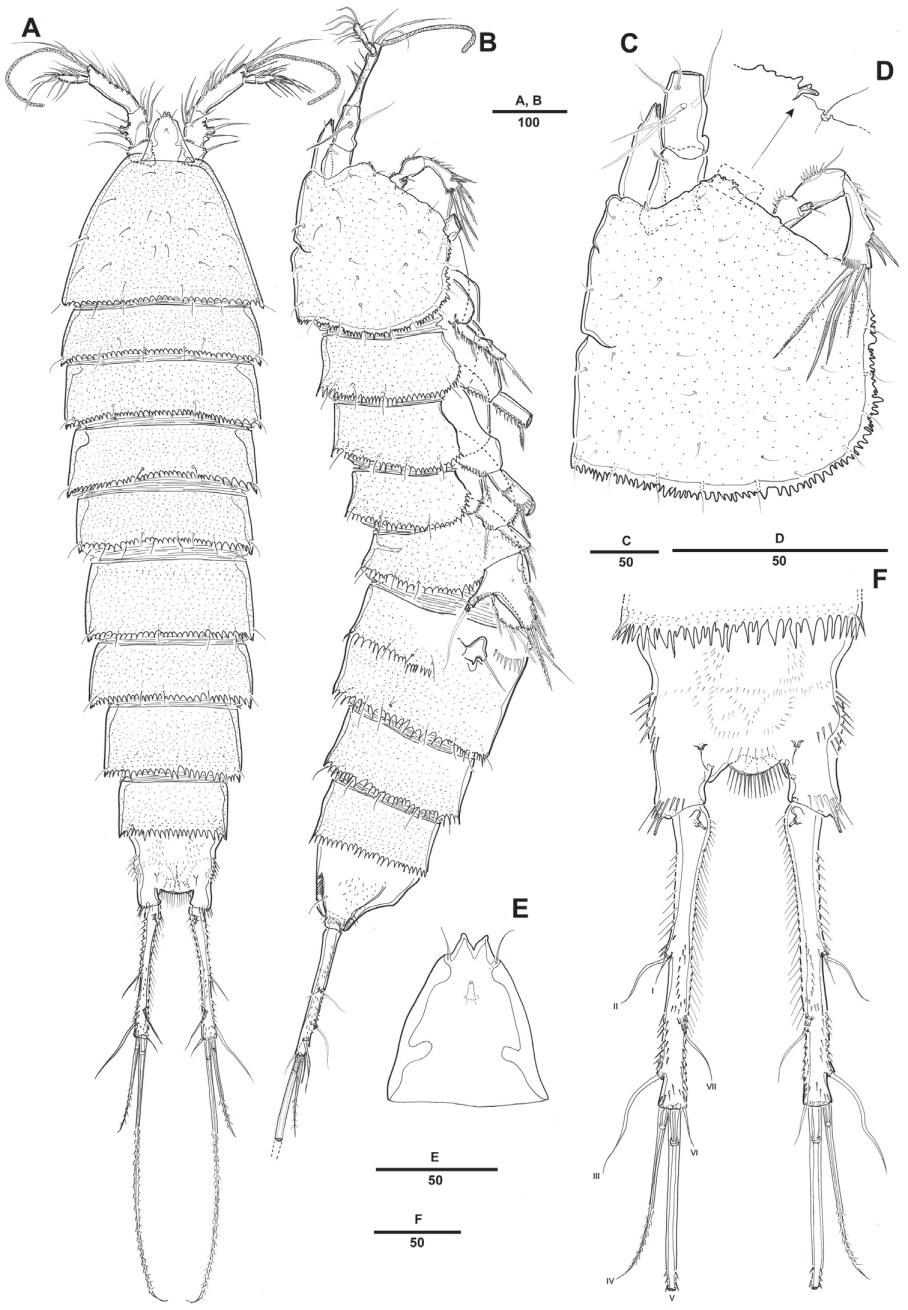
*Pentacletopsyllus montagni* gen. et sp. n. female: **A** habitus, dorsal **B** habitus, lateral **C** cephalothorax, lateral **D** tooth-like process of cephalothorax lateral anterior margin **E** rostrum, dorsal **F** caudal ramus, dorsal.

Figs 1 [Fig F2] [Fig F3] [Fig F4] [Fig F5] –6

##### Type locality.

Gulf of Mexico; 28°43.20'N, 88°20.68'W; depth 1590 m; mud.

##### Material examined.

Holotype: 1♀ (USNM No: 1231418) dissected on 8 slides, from the type locality. Paratypes: 2♀♀ and 1♂ (USNM No's listed in order presented in text: 1231419, 1231420, 1231421) each dissected on 8, 4 and 9 slides respectively, and 10♀♀ and 10♂♂ (USNM No's for specimens in vials (female, male): 1231422, 1231423) in 70% ethanol, vial. Additional samples were deposited in the first author’s collection. All from the type locality, collected by J. G. Baguley on May 2011.

##### Description.

Female. Body (Fig. 1A) elongated. Total body length 1121 µm (n=10; range: 1022–1242 µm, measured from anterior margin of rostrum to posterior margin of caudal rami). Largest width measured at posterior margin of cephalic shield: 234 µm. Urosome narrower than prosome (Fig. 1A). Cephalothorax bell-shaped, with few tegumental sensilla; posterior and lateral margins irregularly serrated (Fig. 1D). Rostrum prominent, triangular; with a pair of sensilla near anterior margin, with bifid tip (Fig. 1E). Pedigerous somites with 1 pair of sensilla on dorsal surface, serrate posteriorly as cephalothorax; pleural areas well developed.

Urosome 5-segmented, comprising P5-bearing somite, genital double-somite and 3 free abdominal somites. All urosomites covered with small spinules dorsally and laterally. Urosomite with serrate posterior and posterolateral margin.

Genital double-somite (Fig. 4C), completely fused ventrally with original segmentation indicated by a transverse surface ridge dorsally and laterally. Genital field located near anterior margin with gonopore and copulatory pore located in median depression. P6 with 1 bare seta on a small protuberance. Anal somite with well-developed rounded operculum bearing row of setules (Fig. 4D).

Caudal rami cylindrical, about 7 times as long as wide, each ramus with 7 setae: setae I-II small, closely set, seta III subapical, setae IV-V bipinnate, seta V longest, seta VI bare and small, seta VII tri-articulate at base; tube pore presented dorsally near proximal inner margin (Fig. 1F).

**Figure 2. F2:**
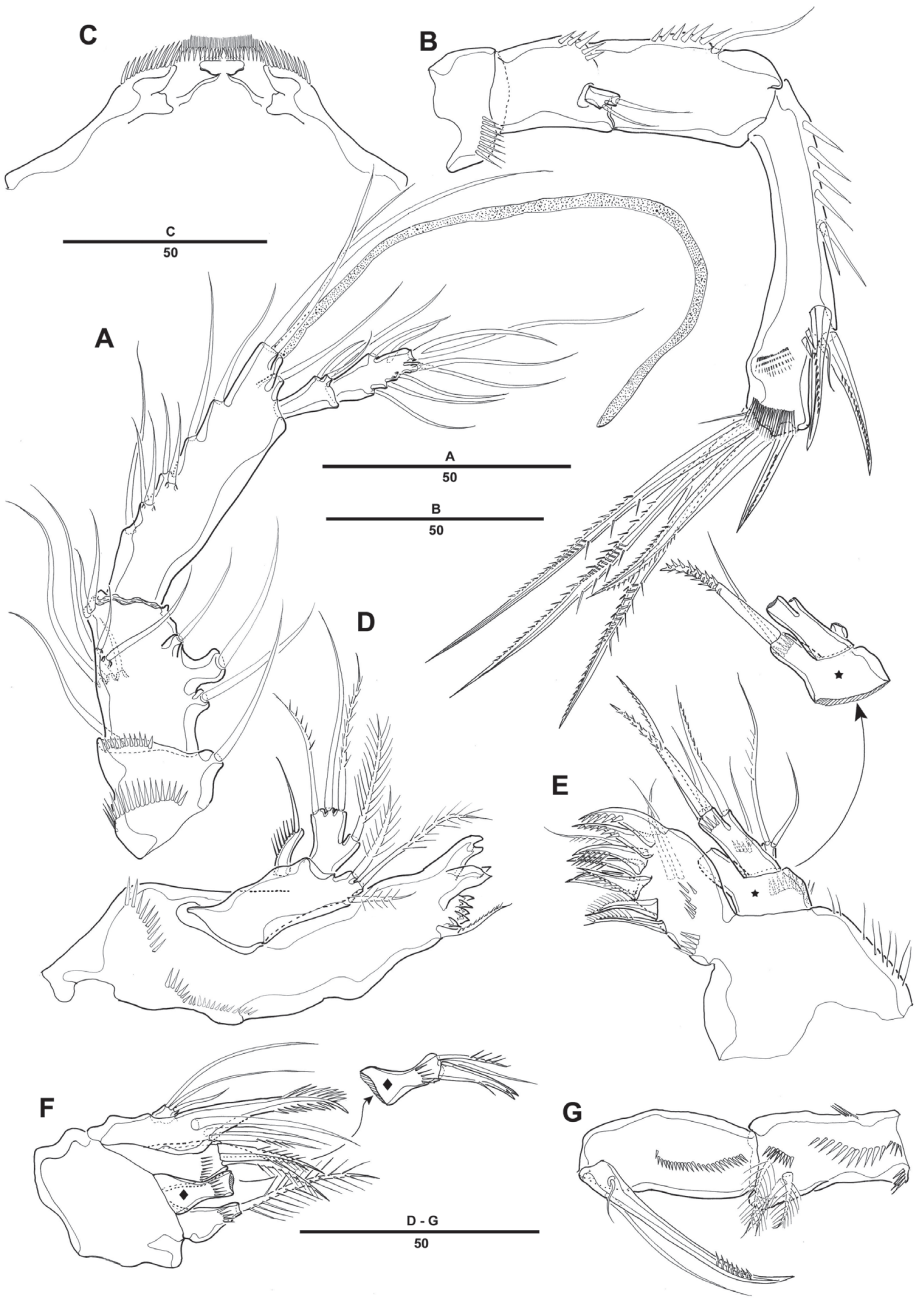
*Pentacletopsyllus montagni* gen. et sp. n. female: **A** antennule, dorsal **B** antenna, dorsal **C** labrum, posterior **D** mandible **E** maxillule (inset showing armature on coxa) **F** maxilla (inset showing armature on middle endite) **G** maxilliped.

Antennule (Fig. 2A) 5-segmented, with well-developed sclerite around base of segment 1. Segment 1 short, with 2 long spinule rows and 1 seta. Segment 2 with 2 outer processes, distal one longer than proximal one, each one bearing one seta distally. Segment 3 longest, with smooth posterior margin. Armature formula: 1-[1], 2-[9], 3-[8 + (1 + ae)], 4-[3], 5-[7 + acrothek]. Apical acrothek consisting of a small aesthetasc fused basally to 2 bare setae.

Antenna (Fig. 2B) 3-segmented, comprising coxa, allobasis and free 1-segmented endopod. Coxa small with spinule row. Allobasis elongated; original segmentation marked by incomplete surface sutures; 2 groups of strong spinules on abexopodal margin; with a long distal abexopodal seta. Exopod small, 1-segmented; with 1 apical and 1 lateral seta. Endopod elongated, with spinules along inner margin; lateral armature consisting of 2 pinnate spines and a minute seta; distal armature consisting of 1 apically curved pinnate spine, 1 bipinnate seta and 3 geniculate setae, the outer-most basally fused to an additional short seta.

Labrum with spinular ornamentation and covered with densely packed setules as in Fig. 2C.

Mandible (Fig. 2D) with large coxa bearing well-developed gnathobase, with 2 strong teeth, several multicuspidate teeth around distal margin and 1 pinnate spine at distal corner; spinules near base of palp. Palp biramous, basis with 3 pinnate setae; endopod with 1 lateral and 3 distal setae; exopod 1-segmented, with 1 apical seta.

Maxillule (Fig. 2E). Precoxa with few spinules near outer margin; arthrite strongly developed, with 2 surface setae and 9 apical spines and setae. Coxa with cylindrical endite bearing 1 naked seta, and 1 curved, pinnate spine. Basis with 2 setae and 1 bipinnate spine apically, and 1pinnate seta along outer margin; with several spinules around inner distal margin and base of endopod. Endopod incorporated in basis and presented by 1 seta. Exopod 1-segmented, with 1 pinnate and 1 naked setae.

Maxilla (Fig. 2F). Syncoxa with 3 endites, each with a short row of spinules. Proximal endite small and with 1 strong pinnate spine. Middle endite produced into pectinate spine and with 2 setae. Distal endite with 3 pinnate setae. Allobasis drawn out into strong, slightly curved, distally pinnate claw, accessory armature consisting of 2 bare and 1 pinnate seta. Endopod small, with 3 naked setae.

Maxilliped (Fig. 2G) comprising syncoxa, basis, and 1-segmented endopod. Syncoxa with 3 plumose setae and several short rows of spinules. Basis with 1 longitudinal row of spinules along palmar margin. Endopodal segment produced into strong and distally pinnate curved claw; accessory armature consisting of 1 long naked seta and 1 small seta at base.

**Figure 3. F3:**
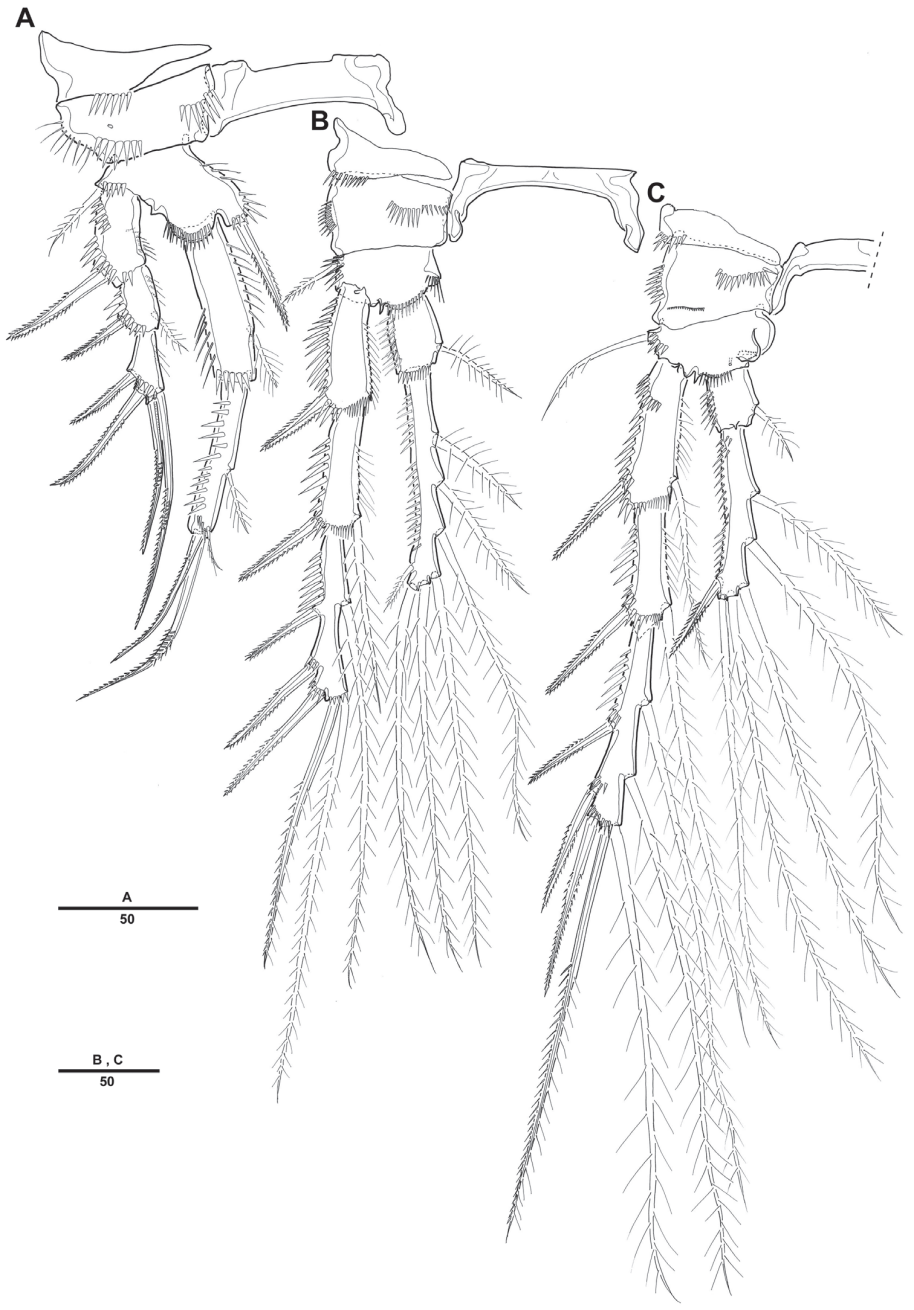
*Pentacletopsyllus montagni* gen. et sp. n. female: **A** P1, anterior **B** P2, anterior **C** P3, anterior.

Swimming legs 1–4 (Fig. 3A, 3B, 3C, 4A) with wide intercoxal sclerite, biramous, endopods 2-segmented, exopods 3-segmented. Coxae and bases with row of spinules along outer margins as illustrated.

**Figure 4. F4:**
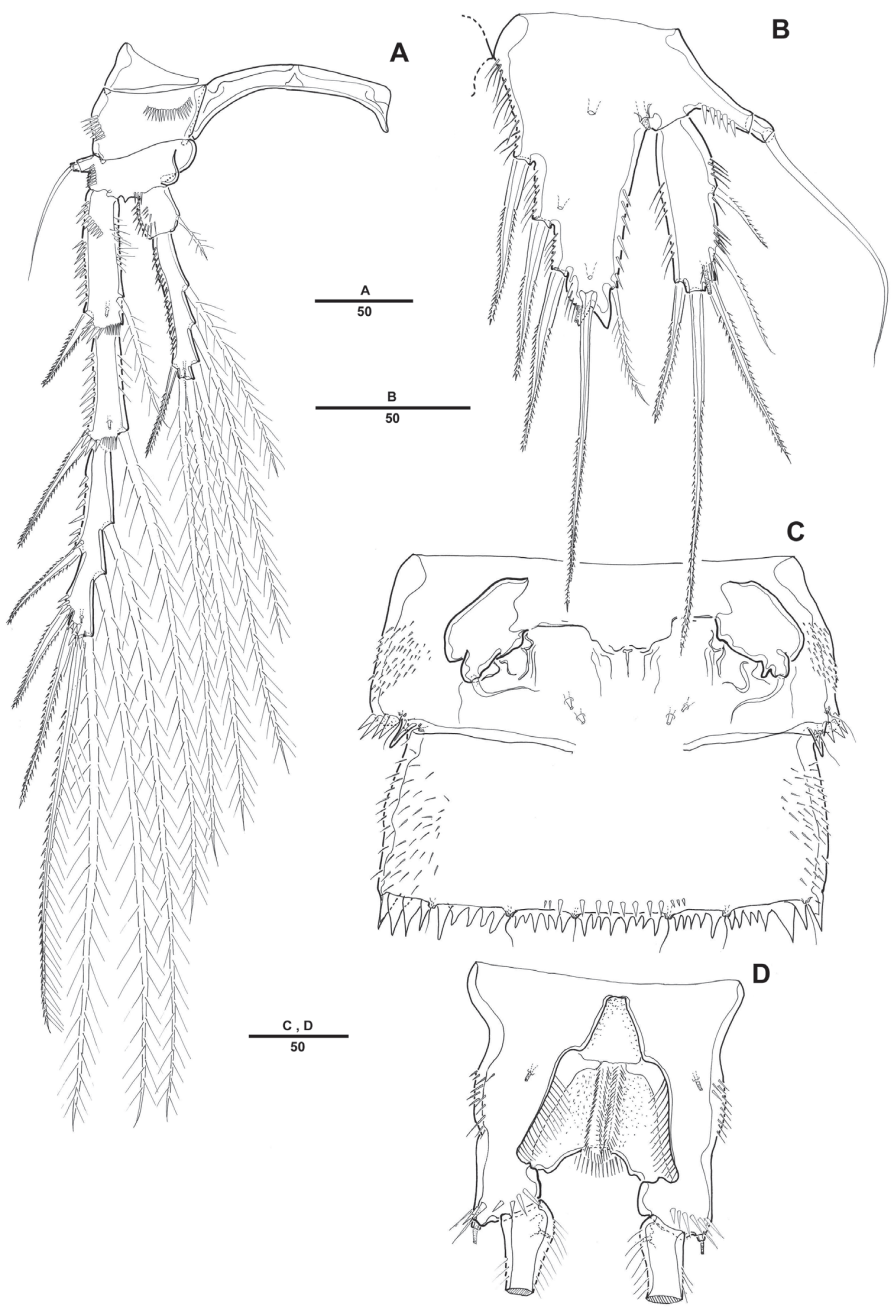
*Pentacletopsyllus montagni* gen. et sp. n. female: **A** P4, anterior **B** P5, anterior **C** genital field, ventral **D** anal somite, ventral.

P1 (Fig. 3A). Coxa large, with inner and outer spinular rows. Basis with strong bipinnate seta on outer margin and bipinnate spine on inner distal surface; inner portion produced as a cylindrical pedestal for endopod. Endopod 2-segmented, prehensile; enp-1 as long as enp-2; enp-1 with one small seta on middle third of inner margin; enp-2 with 2 pinnate inner setae, and 1 pinnate spine and 1 geniculate seta distally. Exopod 3-segmented. Exp-1 and exp-2 with 1 pinnate spine; exp-2 with 1 inner seta; exp-3 with 2 geniculate distal setae and 2 strong spinulose outer spines.

P2-P4 (Figs 3B, 3C, 4A). Coxae and bases with spinular rows along outer margin and anterior surface. Basis wider than long, with pinnate spine (P2-P3) or bare seta (P4), each seta arising from a setophore. Each ramus consisting of 3-segmented exopod and 2-segmented endopod. Armature formula as in generic diagnosis.

P5 (Fig. 4B) with separate exopod and baseoendopod. Baseoendopod longer than wide, forming long biarticulate (not triarticulate) outer setophore bearing the basal seta. Endopodal lobe triangular, with 5 bipinnate setae. Exopod about three times as long as wide, with 1 inner, 1 distal and 3 outer pinnate setae.

##### Description.

**Figure 5. F5:**
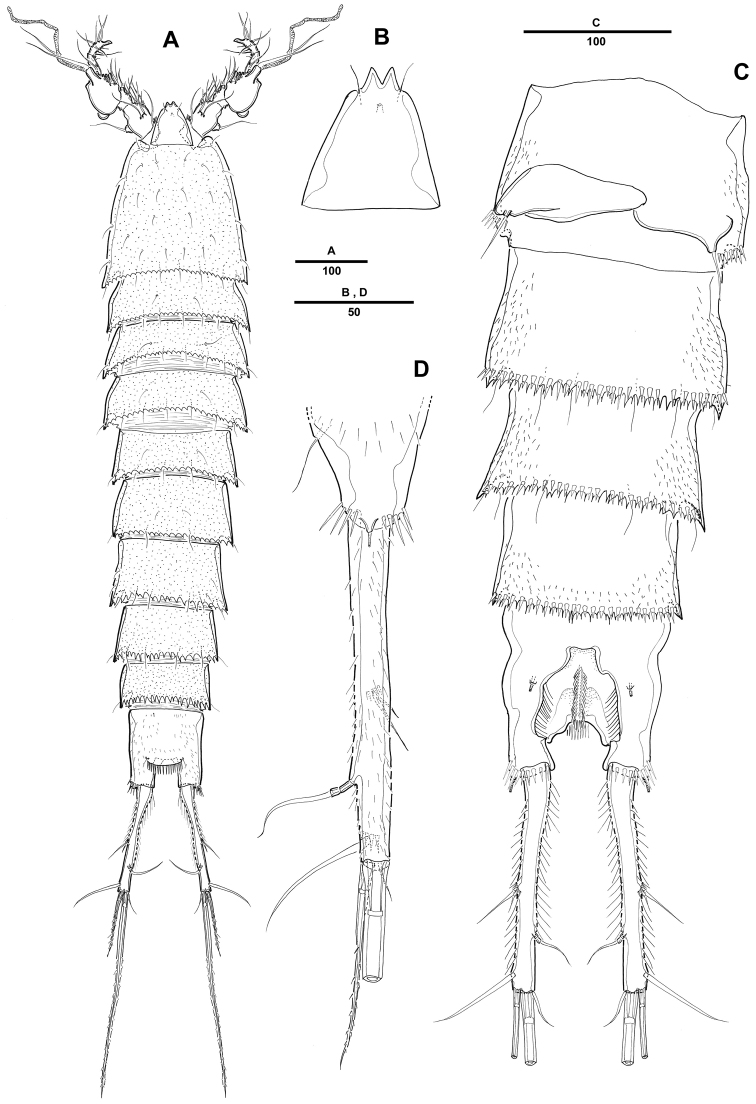
*Pentacletopsyllus montagni* gen. et sp. n. male: **A** habitus, dorsal **B** rostrum, dorsal **C** Urosome (excluding P5-bearing somite), ventral **D** anal somite and left caudal rami, lateral.

Male. Body (Fig. 5A). Male slightly smaller and more slender than in female. Body length 1075 µm (N=10; range: 1007–1132 µm, measured from anterior margin of rostrum to posterior margin of caudal rami). Largest width measured at P2-bearing thoracic somite: 196 µm. Sexual dimorphism in antennule, P2 endopod, P5 and P6.

Prosome (Fig. 5A) 4-segmented, comprising cephalothorax (bearing first pedigerous somite) and 3 free pedigerous somites. Posterior margin of cephalothorax and pedigerous somites with serrated process, with integumental sensilla. Rostrum as in female (Fig. 5B).

Urosome (Fig. 5C) 6-segmented, comprised of P5-bearing somite, genital somite, and 4 free abdominal somites. Urosomite with crenulate posterior margin dorsally and ventrally.

**Figure 6.**
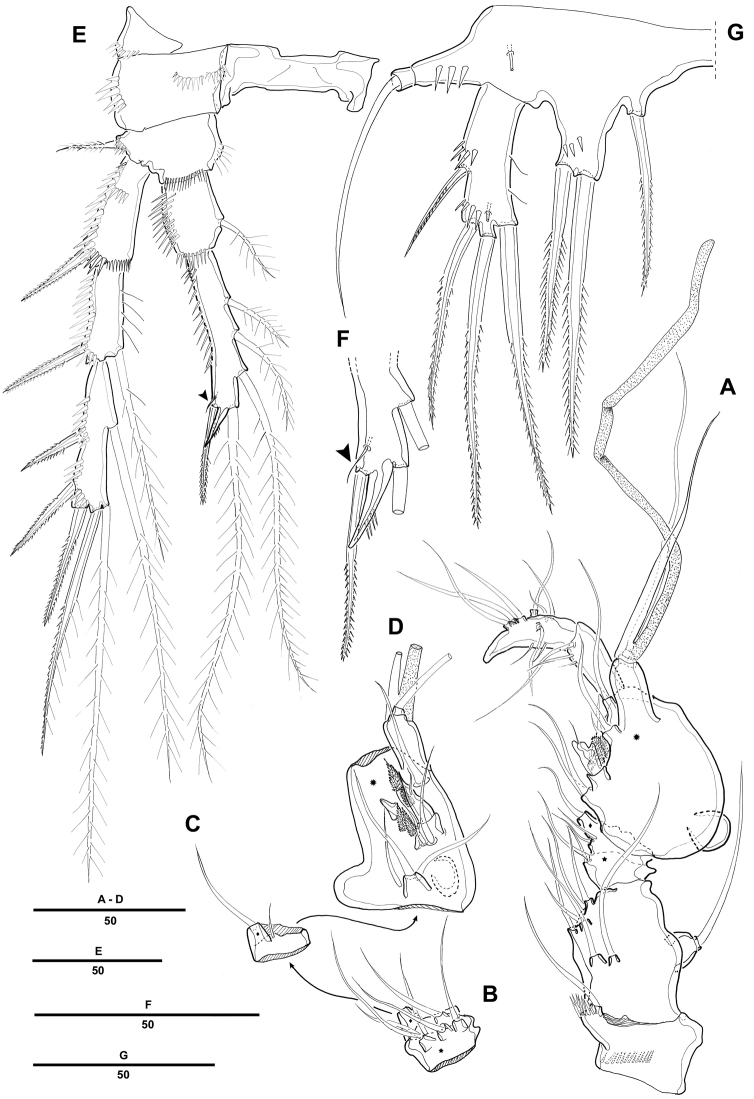
*Pentacletopsyllus montagni* gen. et sp. n. male: **A** antennule, ventral **B** third segment of antennule, anterior **C** fourth segment of antennule, anterior **D** fifth segment of antennule, anterior **E** P4, anterior **F** P4 endopod 3 (arrow indicating reduced outer seta), anterior **G** P5, anterior.

Antennule (Fig. 6A) 7-segmented; subchirocer with geniculation between segments 5 and 6. Segment 1 with 1 row of long spinules along outer distal margin. Segment 2 with 1 cylindrical process along posterior margin, with 1 seta apically. Segment 4 represented by a small sclerite along anterior margin. Segment 5 swollen with large bump along posterior margin. Segment 7 with triangular distal half. Armature formula: 1-[1], 2-[7], 3-[6], 4-[2], 5-[7 + 4 pinnate + 1 modified + (1 + ae)], 6-[3], 7-[7 + acrothek]. Apical acrothek consisting of 2 small naked setae.

P2 (Fig. 6E). Exopod as in female. Endopod modified, 2-segmented; enp-2 with 4 plumose inner setae; inner apical seta fused to segment forming short apophysis; outer apical seta shorter than female; outer seta reduced and presented by minute naked seta (Fig. 6F).

Fifth pair of legs (P5) (Fig. 6G) fused medially. Baseoendopod with outer setophore bearing the basal seta. Endopodal lobe with 1 inner and 2 distal bipinnate setae. Exopod shorter than in female, about three times as long as wide, with 1 inner, 1 distal and 2 outer pinnate setae.

P6 (Fig. 5C) asymmetrical, bearing 1 naked seta on a cylindrical process.

##### Etymology.

The species is named in honor of Dr. Paul Montagna, Endowed Chair for Ecosystem Studies and Modeling at the Harte Research Institute, Texas A&M University Corpus Christi. Dr. Montagna has had a long and distinguished career studying meiofauna, hapacticoid systematics, and marine ecosystem dynamics, particularly in the Gulf of Mexico.

## Discussion

The subfamily Cletopsyllinae of the family Normanellidae was raised to familial rank by Huys and Lee (1999). They provided a new diagnosis and revised the genera of the family, and the family Cletopsyllidae was divided into four genera and nine species. Since then, two new species have been added by Song et al. (2010: *Isocletopsyllus maximus*) and Addis et al. (2011: *Isocletopsyllus sardus*), the family Cletopsyllidae currently includes four genera and 11 species.

Huys and Lee (1999) provided the following morphological diagnosis of the family Cletopsyllidae: (1) body elongated, body somites well defined with dentate or crenulate posterior margins, (2) female antennule 4- or 6-segmented, posterior margin of second segment with two distinct conical process, each bearing an apical seta; apical acrothek in both sexes represented by two setae only, (3) male antennule 7-segmented and typically subchirocerate with geniculation between segments 5 and 6; posterior margin of segment 2 with proximal spinous and distal cylindrical process, (4) antenna exopod 1-segmented and bisetose; endopod with 3 lateral and 6 distal element, (5) P1-P4 biramous with 3-segmented exopod and 2-segmented endopod, (6) baseoendopod of P5 with elongated, tri-articulate setophore, (7) the sexual dimorphism of P2 endopod is the most diagnostic character.

*Pentacletopsyllus* gen. n. is placed in the family Cletopsyllidae with the character sets of the presence of a crenulated posterior margin of body somite, posterior margin of female antennule second segment with 2 distinct conical processes, 1-segmented antenna exopod with 2 setae, armature formula of swimming legs, and P5 with separate exopod and baseoendopod in both sexes and baseoendopod with elongated basal seta. The new genus can be readily identified on the basis of the following three characters:

1. Antennule: The genus *Bathycletopsyllus* Huys & Lee, 1999 has a 6-segmented female antennule and the other genera – *Cletopsyllus* Willey, 1935, *Isocletopsyllus* Huys & Lee, 1999, and *Retrocalcar* Huys & Lee, 1999 – have a 4-segmented antennule. However, the new genus displays a 5-segmented female antennule. Generally the third segment of the antennule has a smooth posterior margin, but a crenulated posterior margin is presented in the genus *Cletopsyllus*. The new genus does not show any modifications as in the genus *Cletopsyllus*. Huys and Lee (1999) mentioned that the second segment of the male antennule has 2 processes along the posterior margin, however the genus *Pentacletopsyllus* has only one process on the posterior margin of antennule, moreover the fifth segment has an additional swelling at the posterior margin.
2. Structure of P1: Normally in the family Cletopsyllidae, P1 exopod is 3-segmented, shorter than endopod; P1 endopod 2-segmented, prehensile, enp-1 at least twice as long as enp-2, and consists of an elongate proximal segment with 1 inner seta. The setation on exopod and endopod of P1 in the genus *Pentacletopsyllus* is the same as above, but the proximal and distal segment of P1 endopod are nearly equal in length.
3. P5: Huys and Lee (1999) mentioned that the baseoendopod of both sexes in the family Cletopsyllidae is characterized by an extremely long extension bearing the outer basal seta, and this setophore is typically tri-articulate. The P5 exopod has six setae in the female and 4-5 setae in the male except for *Cletopsyllus papillifer* Willey, 1935 with seven setae in the female. The new genus has long setophore but not extremely long, and the P5 baseoendopod has a bi-articulate outer setophore in both sexes, and the female P5 has only five setae on the exopod.

Currently, ten species of Cletopsyllidae are known from shallow and sublittoral marine habitats in India (*Retrocalcar secundus* (Nicholls, 1945); *Cletopsyllus bacescui* Marcus, 1976), in Far East Asia (*Retrocalcar sagamiensis* (Itô, 1971); *Isocletopsyllus maximus* Song, Kim & Hwang, 2010), in northeastern America and in Caribbean (*Cletopsyllus papillifer* Willey, 1935; *Retrocalcar brattstroemi* (Geddes, 1981); *Cletopsyllus rotundifera* Fiers, 1986), and in the Mediterranean (*Isocletopsyllus tertius* (Por, 1964); *Isocletopsyllus quartus* (Soyer, 1966); *Isocletopsyllus sardus* Addis, Floris & Carcupino, 2011). Additionally, *Bathycletopsyllus hexarthra* was reported from deep-sea (depth of 460 m) in the Indian Ocean (Huys and Lee 1999), and new genus has been reported in muddy bottom from 1590 m depth in Gulf of Mexico. The family Cletopsyllidae is not common in the marine benthic environment but it is distributed widely throughout the world.

Together with newly described *Pentacletopsyllus montagni*, the 5 genera and 12 species currently recognized as valid in the family Cletopsyllidae can be identified with the specific key given below. It is amended from Huys and Lee (1999) and Wells (2007).

### Key to genera and species of the family Cletopsyllidae

**Table:** 

1	Female antennule 6-segmented	*Bathycletopsyllus hexarthra*
–	Female antennule 5-segmented	*Pentacletopsyllus montagni* sp. n.
–	Female antennule 4 segmented	2
2	Third segment of female antennule with crenulate posterior margin; male fifth leg with 4 setae/spines on exopod	3
–	Third segment of female antennule with smooth posterior margin; male fifth leg with 5 setae/spines on exopod	5
3	P5 endopodal lobe without (male) or with (female) short rounded terminal process; second endopodal segment of first leg with 1 inner seta; rostrum with rounded apex	*Cletopsyllus rotundifera*
–	P5 endopodal lobe of both sexes with long curved terminal process; second endopodal segment of first leg with 2 inner setae; rostrum trifid or bifid at tip	4
4	Inner seta on first endopodal segment of leg 1 inserted at 55% of segment length; endopodal lobe of female fifth leg elongate, rectangular	*Cletopsyllus bacescui*
–	Inner seta on first endopodal segment of leg 1 inserted at 66% of segment length; endopodal lobe of female fifth leg short, triangular	*Cletopsyllus papillifer*
5	Female caudal rami with outer proximal margin produced as a lobate expansion bearing a spur-like process posteriorly and secondary process dorsally	6
–	Female caudal rami without lobate expansion; ramus markedly longer in male	8
6	Second endopodal segment of first leg with 1 inner seta	*Retrocalcar secundus*
–	Second endopodal segment of first leg with 2 inner setae	7
7	Caudal ramus 3.6 (male) and 6.5 (female) times as long as greatest width; outer spines on third exopodal segment of leg 3 modified in male	*Retrocalcar brattstroemi*
–	Caudal ramus 5.3 (male) and 9.0 (female) times as long as greatest width; outer spines on third exopodal segment of leg 3 not modified in male	*Retrocalcar sagamiensis*
8	Caudal ramus less than 6 times as long as wide; rostrum trifid at tip	9
–	Caudal ramus more than 7 times as long as wide; rostrum bifid at tip	10
9	First antennular segment without outer process; caudal ramus without expanded inner border	*Isocletopsyllus tertius*
–	First antennular segment with outer process; caudal ramus with expanded inner border	*Isocletopsyllus maximus*
10	Antennary exopod with 2 setae of equal length; distal margin of basis of leg 1 without spinous process between exopod and endopod	*Isocletopsyllus quartus*
–	Antennary exopod with 2 setae of different length; distal margin of basis of leg 1 with spinous process between exopod and endopod	*Isocletopsyllus sardus*

## Supplementary Material

XML Treatment for Pentacletopsyllus

XML Treatment for Pentacletopsyllus montagni

## References

[B1] Addis A Floris A Carpucino M (2011) A new species of the genus *Isocletopsyllus* (Harpacticoida, Cletopsyllidae). Meiofauna Marina 19(1): 166-171

[B2] Baguley JG Montagna PA Lee W Hyde LJ Rowe GT (2006) Spatial and bathymetric trends in Harpacticoida (Copepoda) community structure in the Northern Gulf of Mexico deep-sea. Journal of Experimental Marine Biology and Ecology 330(1): 327-341. 10.1016/j.jembe.2005.12.037

[B3] Boxshall GA Halsey SH (2004) An introduction to copepod diversity. Ray Society, London, 966 pp.

[B4] Burgess R (2001) An improved protocol for separating meiofauna from sediments using colloidal silica sols. Marine Ecology Progress Series 214: 161-165. 10.3354/meps214161

[B5] Fiers F (1986) Harpacticoid copepods from the West Indian Islands: Laophontidae (Copepoda, Harpacticoida). Bijdragen tot de Dierkunde 56(1): 132-164

[B6] Geddess DC (1981) Marine biological investigations in the Bahamas 21. A new species of Cletopsyllus (Copepoda, Harpacticoida). Sarsia 66(4): 287-291

[B7] Haedrich RL Devine JA Kendall VJ (2008) Predictors of species richness in the deep-benthic fauna of the northern Gulf of Mexico. Deep Sea Research Part II: Topical Studies in Oceanography 55(24): 2650-2656. 10.1016/j.dsr2.2008.09.003

[B8] Huys R Gee JM Moore CG Hamond R (1996) Marine and brackish water harpacticoid copepods part 1: keys and notes for identification of the species. The Linnean Society of London, London, 352 pp.

[B9] Huys R Lee W (1999) On the relationships of the Normanellidae and the recognition of Cletopsyllidae grad. nov. (Copepoda, Harpacticoida). Zoologischer Anzeiger 237(4): 267-290

[B10] Huys R Willems K (1989) *Laophontopsis* Sars and the taxonomic concept of the Normanellinae (Copepoda: Harpacticoida): a revision. Bijdragen tot de Dierkunde 59(4): 203-277

[B11] Itô T (1971) A new species of the genus Cletopsyllus from Sagami Bay (Harpacticoida). Annotationes Zoologicae Japonenses 44(2): 117-124

[B12] Lee W Montagna PA Han MS (2003) Three new species of the genus Normanella Brady (Copepoda: Harpacticoida) from the Gulf of Mexico. Journal of natural History 37(10): 1219-1245. 10.1080/00222930110109064

[B13] Marcus A (1976) Contributions to the study of the genus Cletopsyllus Willey 1935 (Copepoda, Harpacticoida) from the Indian Ocean. Travaux du Muséum d’histoire naturelle “Grigore Antipa” 17: 39-49

[B14] Montagna PA Baguley JG Cooksey C Hartwell I Hyde LJ Kalke RD Kracker LM Reuscher M Rhodes ACE (2013) Deep-sea benthic footprint of the Deepwater Horizon Blowout. PLoS ONE 8(8): . 10.1371/journal.pone.0070540 10.1371/journal.pone.0070540 PMC3737147 23950956

[B15] Nicholls AG (1945) Marine Copepoda from Western Australia. III. Littoral harpacticoids from Port Denison. Journal of the Royal Society of Western Australia 29: 1-16

[B16] Por FD (1964) A study of the Levantine and Pontic Harpacticoida (Crustacea, Copepoda). Zoologische Verhandelingen 64: 1-128

[B17] Song SJ Kim W Hwang UW (2010) First record of the family Cletopsyllidae (Copepoda: Harpacticoida) from Korean waters, with description of a new species. Animal Cells and Systems 14(4): 351-360. 10.1080/19768354.2010.525801

[B18] Soyer J (1966) Copépodes Harpacticoïdes de Banyuls-sur-Mer. 3. Quelques formes du coralligène. Vie Milieu 17(1-B): 303–344

[B19] Vervoort W (1964) Free-living Copepoda from Ifaluk Atoll, in the Caroline Islands: with notes on related species. Smithsonian Institution, Washington, 431 pp.

[B20] Wells JBJ (2007) An annotated checklist and keys to the species of Copepoda Harpacticoida (Crustacea). Zootaxa 1568: 1-872

[B21] Willey A (1935) IV.-Harpacticoid Copepoda from Bermuda.-Part II. Journal of Natural History Series 10, 15(85): 50–100

